# The Prevalence of Histopathological Features of Pneumonia in Goats with Symptomatic Caprine Arthritis-Encephalitis

**DOI:** 10.3390/pathogens11060629

**Published:** 2022-05-30

**Authors:** Agata Moroz, Michał Czopowicz, Małgorzata Sobczak-Filipiak, Izabella Dolka, Magdalena Rzewuska, Magdalena Kizerwetter-Świda, Dorota Chrobak-Chmiel, Marcin Mickiewicz, Lucjan Witkowski, Olga Szaluś-Jordanow, Tomasz Nalbert, Adrian Valentin Potârniche, Karolina Barszcz, Iwona Markowska-Daniel, Ryszard Puchała, Emilia Bagnicka, Jarosław Kaba

**Affiliations:** 1 Division of Veterinary Epidemiology and Economics, Institute of Veterinary Medicine, Warsaw University of Life Sciences–SGGW, Nowoursynowska 159c, 02-776 Warsaw, Poland; agata_moroz@sggw.edu.pl (A.M.); marcin_mickiewicz@sggw.edu.pl (M.M.); lucjan_witkowski@sggw.edu.pl (L.W.); tomasz_nalbert@sggw.edu.pl (T.N.); iwona_markowska_daniel@sggw.edu.pl (I.M.-D.); 2 Department of Pathology and Veterinary Diagnostics, Institute of Veterinary Medicine, Warsaw University of Life Sciences–SGGW, Nowoursynowska 159c, 02-776 Warsaw, Poland; malgorzata_sobczak_filipiak@sggw.edu.pl (M.S.-F.); izabella_dolka@sggw.edu.pl (I.D.); 3 Department of Preclinical Sciences, Institute of Veterinary Medicine, Warsaw University of Life Sciences–SGGW, Ciszewskiego 8, 02-786 Warsaw, Poland; magdalena_rzewuska@sggw.edu.pl (M.R.); magdalena_kizerwetter_swida@sggw.edu.pl (M.K.-Ś.); dorota_chrobak@sggw.edu.pl (D.C.-C.); 4 Department of Small Animal Diseases with Clinic, Institute of Veterinary Medicine, Warsaw University of Life Sciences–SGGW, Nowoursynowska 159c, 02-776 Warsaw, Poland; olga_szalus_jordanow@sggw.edu.pl; 5 Department of Infectious Diseases, Faculty of Veterinary Medicine, University of Agricultural Sciences and Veterinary Medicine of Cluj-Napoca, Mănăștur Str. 3-5, 400372 Cluj, Romania; adrian.potarniche@usamvcluj.ro; 6 Department of Morphological Sciences, Institute of Veterinary Medicine, Warsaw University of Life Sciences–SGGW, Nowoursynowska 159c, 02-776 Warsaw, Poland; karolina_barszcz@sggw.edu.pl; 7 Applied Physiology Unit, Military Institute of Hygiene and Epidemiology, Kozielska 4, 01-163 Warsaw, Poland; rapuchala@gmail.com; 8 Institute of Genetics and Animal Biotechnology, Polish Academy of Sciences, Postępu 36A, Jastrzębiec, 02-552 Magdalenka, Poland; e.bagnicka@igbzpan.pl

**Keywords:** CAE, histopathology, immunohistochemistry, interstitial pneumonia, *Mannheimia*, small ruminant lentivirus

## Abstract

Chronic interstitial pneumonia (CIP) is a main pathology of sheep infected with small ruminant lentivirus (SRLV). Caprine arthritis-encephalitis (CAE) is caused by the same pathogen; however, the presence of CIP has been only occasionally reported in SRLV-infected goats. We carried out a cross-sectional study to determine the prevalence of histopathological lesions indicative of CIP in goats with symptomatic CAE, and to investigate whether CIP was associated with a higher prevalence of other types of pneumonia (purulent bronchopneumonia, fibrinous pleuropneumonia) or bacterial infections. Lung specimens and bronchial swabs were collected for histopathological and bacteriological examination, respectively, from 116 goats from a CAE-affected herd. All goats were euthanized due to severe clinical signs of CAE. The goats were seropositive for SRLV infection in two different ELISAs and the presence of SRLV antigen in the lung tissue was confirmed by immunohistochemistry. Histopathologically, pneumonia of any type was confirmed in 82 goats (70.7%) and CIP was present in 67 goats (57.8%). In most goats, the severity of the histopathological features of pneumonia was mild. Bacteria were detected in bronchial swabs from 73 goats (62.9%). CIP proved to be significantly positively linked to the occurrence of purulent bronchopneumonia (*p* < 0.001), fibrinous pleuropneumonia (*p* = 0.001), and of the infection of lungs with bacteria capable of causing pneumonia (*p* = 0.050). The causal character of these associations should be considered and warrants further investigation.

## 1. Introduction

Caprine arthritis-encephalitis (CAE) is a goat disease caused by a small ruminant lentivirus (SRLV). Its counterpart in sheep is maedi-visna disease. SRLV is a heterogenous viral species comprising at least four genotypes and many genetic subtypes with genotype A (traditionally referred to as maedi-visna virus (MVV)-like) and B (CAEV-like) distributed worldwide [1]. Even though it has so far been well-evidenced that SRLV easily crosses the interspecies barrier between sheep and goats [2,3,4,5,6,7] and both diseases demonstrate a similar chronic progressive course with a long incubation period, the two small ruminant species tend to show distinct clinical signs [8]. A progressive arthritis in goats and a progressive interstitial pneumonia in sheep are the main clinical manifestations of SRLV infection, usually accompanied by emaciation and sometimes by indurative mastitis [9].

Maedi-visna disease is widespread in many European countries, with Greek and Spanish sheep populations being the most extensively affected [10]. In Poland, the flock-level seroprevalence ranges from <1% to 50% with the average individual-level seroprevalence of 15% [10,11]. Despite the fact that for at least two decades genotype A (MVV-like) has been detected in goat populations in many countries [12] including Poland [13,14,15], the knowledge of the occurrence of pulmonary lesions in the course of CAE is very limited. In fact, only some early studies have indicated and described maedi-visna-like pulmonary lesions in SRLV-infected goats [16,17,18,19]. Thenceforth, SRLV infection has only been mentioned as a potential but infrequent cause of interstitial pneumonia in goats [8]. On the other hand, interstitial pneumonia has also been observed in goats infected with genotype B [20], which is traditionally regarded as a caprine SRLV genotype and has also been shown to occur throughout Poland [13,14,15].

In Poland, CAE is a widespread disease, with herd-level seroprevalence exceeding 70% [21], and goats with clinical signs of arthritis and emaciation are present in most infected herds [22]. Despite this fact, farmers decide not to introduce test-and-cull control programs mainly due to financial constraints. Some herds practice early weaning of kids from infected does, usually with only moderate successes [23]. Most farmers restrict themselves to culling goats showing clinical signs of CAE. One cross-sectional questionnaire study carried out in Polish goat herds indicated a significant relationship between the occurrence of SRLV infection and respiratory signs at a herd-level, although the link with arthritis and emaciation was much stronger [24]. For a few years, we cooperated with a large goat farm extensively affected by CAE, caused by SRLV genotype A, and we assisted in the elimination of a number of severely arthritic and emaciated goats. Therefore, we decided to use these goats to determine the prevalence of interstitial pneumonia in goats with symptomatic CAE and to investigate if interstitial pneumonia was associated with a higher prevalence of other types of pneumonia and bacterial infections of the lungs.

## 2. Results

### 2.1. Serological Status

Of 122 euthanized, goats 116 goats were seropositive for SRLV in two different ELISAs (ELISA based on the whole virus antigen (wELISA) and ELISA based on recombinant transmembrane (TM) and capsid (CA) antigens (TM/CA-ELISA)) and were hence enrolled in the further analyses. Their age ranged from 1 to 14 years with a median (IQR) of 6 (4 to 8) years. Given a high diagnostic specificity of both wELISA and TM/CA-ELISA and a very high true prevalence of SRLV infection in the herd from which the goats came, the diagnosis based on a double positive result was very likely to be true (positive predictive value of >99.9%). All 116 goats tested seropositive for PIV-3 infection and 70/116 goats for caseous lymphadenitis (CLA), which yielded a seroprevalence of 100% (CI 95%: 96.8–100%) and 60.3% (CI 95%: 51.2–68.8%), respectively.

### 2.2. Gross and Histopathological Lesions

**Figure 1:**
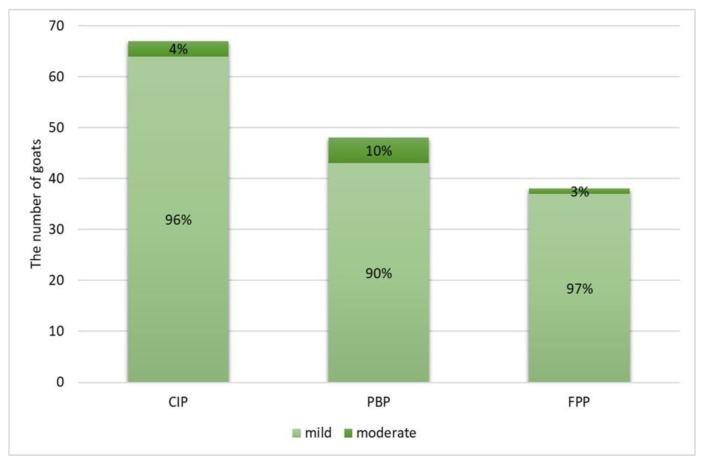
The severity of three pneumonia types in a study goat population: chronic interstitial pneumonia (CIP), purulent bronchopneumonia (PBP), and fibrinous pleuropneumonia (FPP).

None of goats showed any gross pulmonary lesions indicative of SRLV-associated pneumonia. Microscopic examination of the lung specimens revealed that pneumonia of any type was observed in 82/116 goats (70.7%; CI 95%: 61.8–78.2%). CIP was present in 67/116 goats (57.8%; CI 95%: 48.7–66.4%), PBP in 48/116 goats (41.4%; CI 95%: 32.8–50.5%), and FPP in 38/116 goats (32.8%; CI 95%: 24.9–41.7%). Pneumonia features were classified as mild in most goats, moderate only in a few goats, and severe in none (Figure 1).

**Figure 2:**
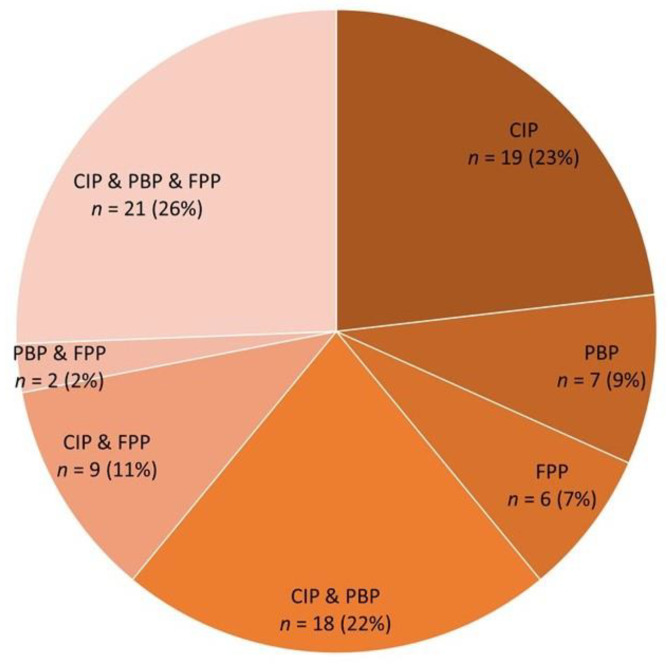
The prevalence of three pneumonia types in a study goat population: chronic interstitial pneumonia (CIP), purulent bronchopneumonia (PBP), and fibrinous pleuropneumonia (FPP).

A single pattern was observed in 32 goats (27.6% of all goats, and 39% of 82 goats with pneumonia confirmed histopathologically), of which most (*n* = 19, 59%) had CIP. A mixed pattern was observed in 50 goats, and only in 2 of them was CIP not involved (Figure 2).

**Figure 3:**
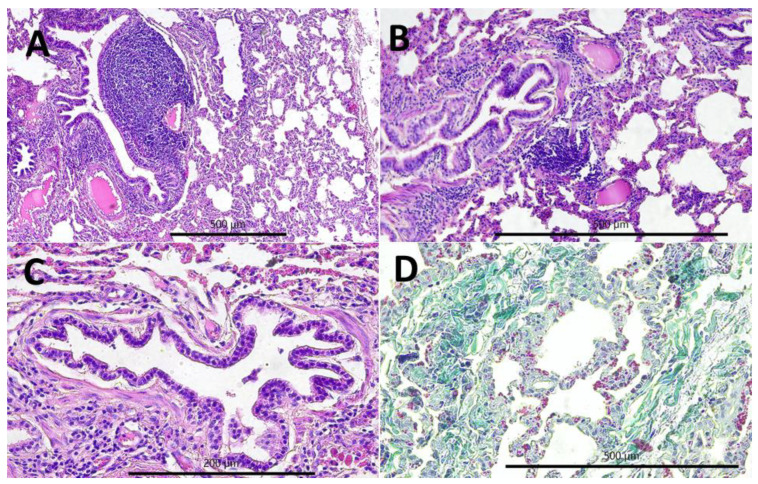
Histopathological lesions observed in the chronic interstitial pneumonia (CIP): (**A**) lymphatic nodules hyperplasia (peribronchial and perivascular), interstitial (i.e., peribronchiolar and perialveolar) inflammatory infiltrates composed of macrophages and lymphocytes, interalveolar septum thickening, 100×, H-E; (**B**) interstitial (i.e., peribronchiolar and perialveolar) inflammatory infiltrates composed of macrophages and lymphocytes, interalveolar septum thickening, 200×, H-E; (**C**) smooth muscle hyperplasia in the walls of the bronchioles, 400×, H-E; and (**D**) interstitial connective tissue hyperplasia, 200×, Masson.

**Figure 4:**
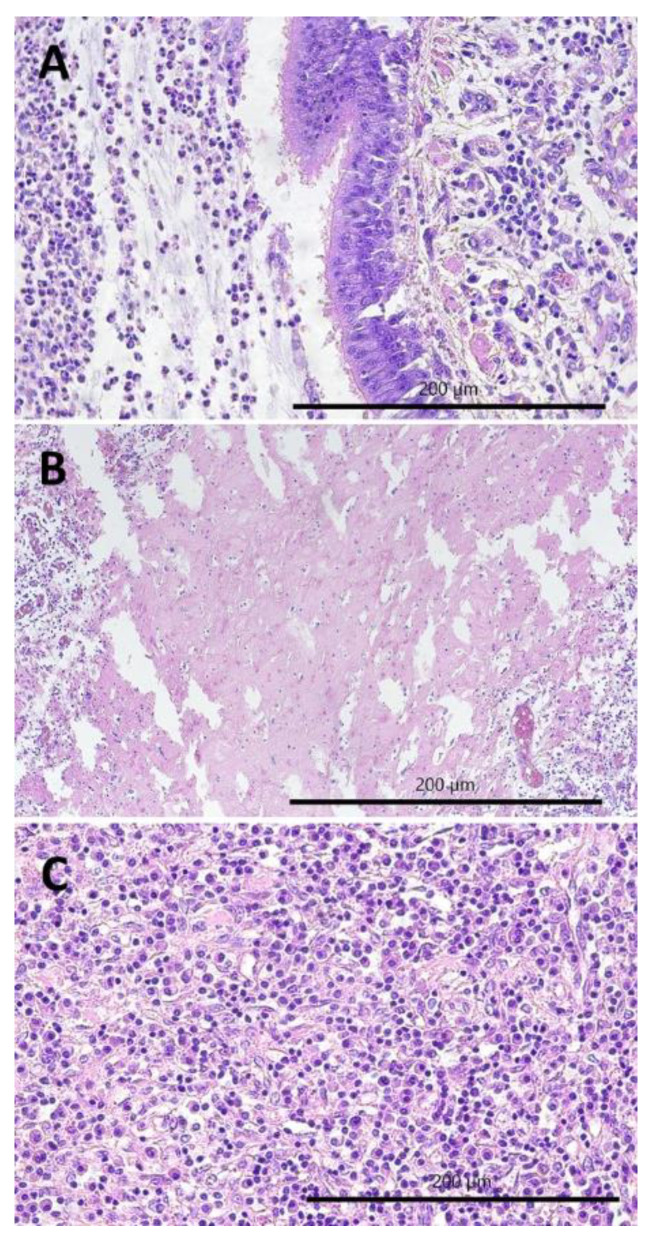
Histopathological lesions observed in the purulent bronchopneumonia (PBP), 400×, H-E: (**A**) peribronchial inflammatory infiltrates composed of neutrophils and lymphocytes, purulent exudate in the lumen of bronchus; (**B**) focal necrosis of lung parenchyma; and (**C**) focal atelectasis.

**Figure 5:**
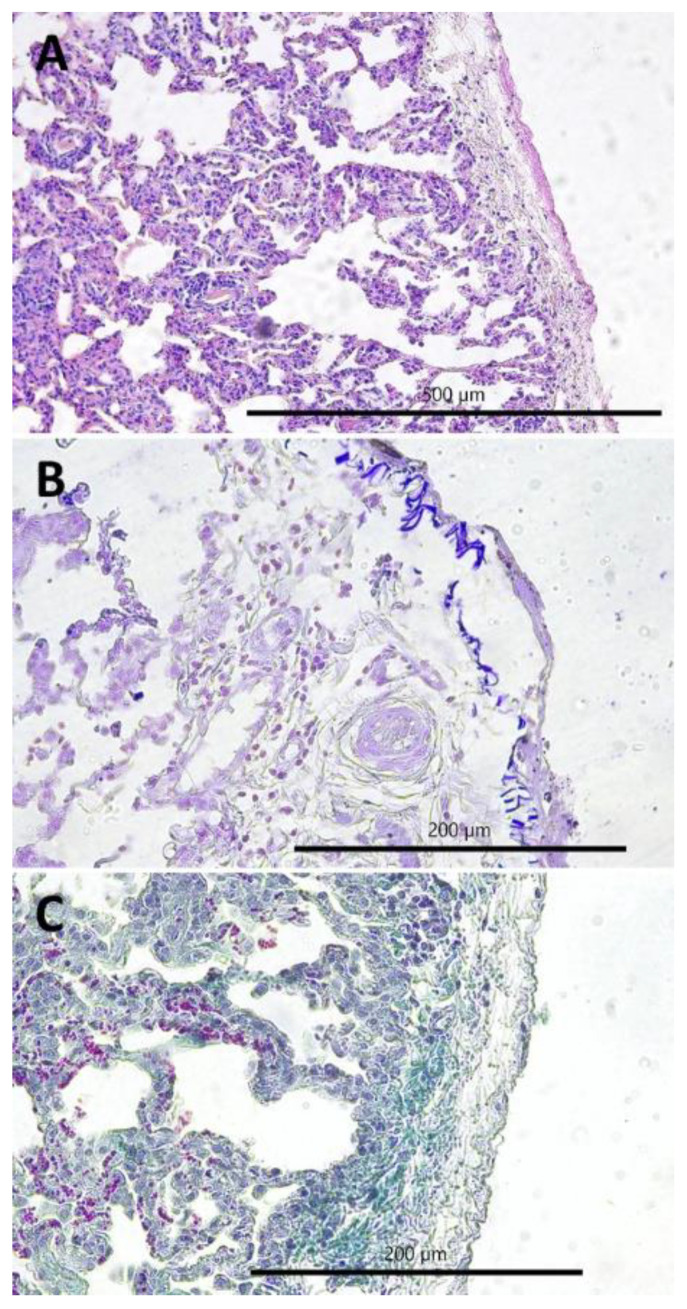
Histopathological lesions observed in the fibrinous pleuropneumonia pattern (FPP): (**A**) subpleural accumulation of fibrin, 200×, H-E; (**B**) subpleural accumulation of fibrin, 400×, Weigert; and (**C**) subpleural connective tissue hyperplasia, 400×, Masson.

The most commonly observed histopathological lesions were inflammatory infiltrates: interstitial (Figure 3A,B) in CIP and peribronchial and perivascular in PBP (Figure 4A). In CIP, they were followed by lymphatic nodules formation (Figure 3B) and interstitial connective tissue hyperplasia (Figure 3D), which were observed in more than half of the goats with this type of pneumonia, whereas smooth muscle hyperplasia (Figure 3C), interstitial septum thickening, and pneumocyte hyperplasia were much less common (Figure 3B). In PBP, other lesions such as focal emphysema and necrosis (Figure 4B,C) occurred in less than one-fifth of the goats. In FPP, subpleural connective tissue hyperplasia (Figure 5A) was observed most often and fibrin accumulation (Figure 5B,C) was present in less than one-fourth of the goats with this type of pneumonia (Table 1).

**Figure 6:**
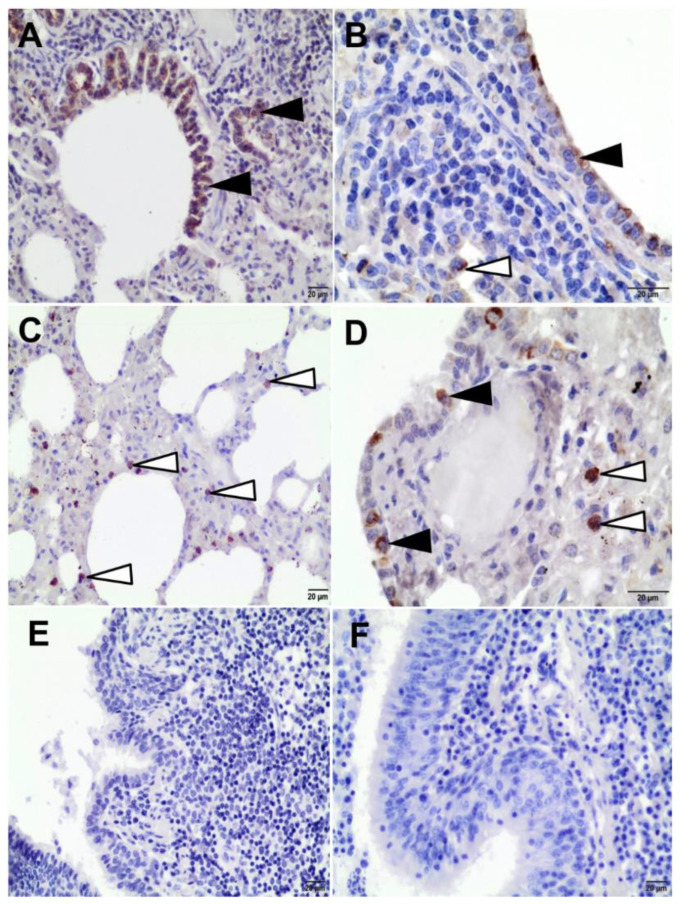
Representative images of immunohistochemistry (IHC) examination of caprine lung samples. The immunoreactivity for small ruminant lentivirus (SRLV) was developed with DAB chromogen (brown precipitate in the cell’s cytoplasm) and counterstained with Mayer’s hematoxylin (**A**–**D**) positive immunostaining and (**E**,**F**) negative controls: (**A**) the bronchiole lined by SRLV-positive epithelial cells (black arrowheads), 200×; (**B**) the SRLV-positive bronchial epithelial cells (black arrowhead) and a macrophage (white arrowhead) in the peribronchial inflammatory cells, 400×; (**C**) the SRLV-positive macrophages scattered in the thickened alveolar septa (white arrowheads), 200×; (**D**) the positive immunostaining of SRLV in the cytoplasm of the bronchial epithelial cells (black arrowhead) and macrophages (white arrowhead), 400×; (**E**) a negative tissue control, 200×; and (**F**) an isotype control, 200×.

IHC was positive in 28/30 examined goats (93.3%). In 23/28 IHC-positive goats (82.1%), IHC staining was found both in bronchial epithelial cells and in macrophages located in the peribronchial interstitial tissue and in interalveolar septa (Figure 6B,D). In 3/28 goats (10.7%), IHC staining was present only in bronchial epithelial cells (Figure 6A), and, in 2/28 goats (7.2%), only in macrophages (Figure 6C). No specific labelling was observed in the SRLV-negative control (Figure 6E,F). The 2 goats that tested negative in IHC staining did not show histopathological lesions indicative of any type of pneumonia.

### 2.3. Bacterial Infections

Bacteria were detected in bronchial swabs from 73/116 goats (62.9%; CI 95%: 53.9–71.2%): a single bacterial species was isolated from 57/73 goats (78.1%) and two isolates from 16/73 goats (21.9%). Potential etiological agents of bacterial pneumonia were isolated from 40/116 goats (34.5%; CI 95%: 26.5–43.5%). Accidental/commensal microorganisms were detected in 33 goats. The most prevalent bacteria were Enterobacterales (mostly *Escherichia coli*), *Trueperella pyogenes*, Pasteurellaceae *(Mannheimia hemolytica* and *Pasteurella multocida*, virtually equally often), and staphylococci (mostly *Staphylococcus aureus*) (Table 2). *Corynebacterium pseudotuberculosis* was isolated from 6 goats and all of them were seropositive for CLA.

### 2.4. Relationship between Chronic Interstitial Pneumonia and other Respiratory System-Associated Conditions

CIP proved to be significantly positively linked to the occurrence of PBP (*p* < 0.001) and FPP (*p* = 0.001), which were 3–4-fold more likely in goats with than without CIP. CIP also appeared to be a potential risk factor for the infection of lungs with bacteria capable of causing pneumonia. On the other hand, it was not significantly related to CLA (neither seropositive status nor the presence of the etiological agent), the presence of accidental/commensal microorganisms in the lungs, or the concentration of antibodies against PIV-3 (Table 3).

## 3. Discussion

Our study shows that histopathological features of CIP were present in roughly 50% to 60% of the goats with symptomatic CAE caused by SRLV genotype A. Although they were mild in most of these goats, they appeared to be significantly linked to the occurrence of other types of pneumonia as well as bacterial infections of the lungs.

Lower respiratory tract and lung pathologies are a common health problem in goats [8]; however, their prevalence is vastly linked to the environmental factors and management system and varies considerably among geographical regions [25]. Few disease surveys have been published on the prevalence of pneumonia in goats, and they come from outside Europe. A large-scale survey on the prevalence of pneumonia in 374 slaughtered goats in Ethiopia revealed histopathological features of pneumonia in roughly 17% of them, and 30% of these animals had lesions indicating interstitial pneumonia [26]. On the other hand, the prevalence of lung lesions on gross examination in over 1.5 thousand slaughtered goats was less than 3% [27]. In light of these results, roughly 60% of goats with microscopic lesions indicative of CIP, 40% with PBP, and 30% with FPP appears to be a high proportion. Obviously, the goats enrolled in our study cannot be considered representative for the Polish goat population as they were severely affected by CAE. On the other hand, in Polish goats, respiratory signs have been observed in over 80% of goat herds and this percentage increased during the first decade of the 21st century along with an increasing tendency to keep goats in barns without grazing [28]. This may indicate that respiratory tract pathologies are generally widespread in Polish goat population.

CIP is the leading pathology in the course of maedi-visna disease [29] and its severity correlates with peripheral provirus levels [30]. Although progressive arthritis is the main clinical manifestation of CAE, several studies and case reports have shown that interstitial pneumonia also develops in SRLV-infected goats [16,17,18,19,20,31]. Nevertheless, its prevalence in SRLV-infected goats has never been investigated in any large-scale disease survey. The results of our cross-sectional study indicate that CIP develops in a relatively high proportion of arthritic goats. This high prevalence may even be underestimated because we examined histologically only specimens from the cranial lobe, whereas cranioventral or caudal lobes appear to most often contain SRLV-associated lesions [32]. In a large-scale abattoir survey from Ethiopia, roughly 50% of slaughtered sheep and 30% of slaughtered goats with pneumonia in gross examination had histopathological lesions indicative of interstitial pneumonia [26]. The status of goats with respect to SRLV was not evaluated in this study, and the epidemiological situation of the population of 51 million Ethiopian goats is unknown; however, maedi-visna is present and likely widespread in the population of 40 million Ethiopian sheep [33,34,35]. Although this implies that most of the interstitial lesions revealed in this study could have been linked with SRLV infection, it must be underscored that histopathology can provide only a preliminary estimation of SRLV-associated pulmonary pathologies and definitive diagnosis requires the use of pathogen-specific modalities. A recent study from Iran revealed that a small proportion of microscopic lesions suggestive of maedi-visna occurred in sheep truly infected with SRLV [36]. Similar interstitial lesions may be produced, e.g., by infections with *Mycoplasma* spp., which tend to elude routine bacteriological diagnostics [37,38,39,40]. Goats are considered more susceptible to mycoplasmal infections than sheep [41], and *M. ovipneumoniae* seems to be the most prevalent species in sheep and goats in Europe [42,43], with *M. arginine* [44] and *M. capricolum* [45] occasionally detected in pulmonary lesions. In our study, we did not perform any diagnostic tests for these pathogens. Therefore, the potential concomitant role of various *Mycoplasma* spp. needs to be taken into account as an alternative explanation of the observed interstitial lesions, especially given the fact that cranio-ventral lobes of the lungs are considered a typical location for mycoplasmal pneumonia [46]. The occurrence of *Mycoplasma* spp. infections in Polish small ruminant populations has never been investigated; however, serosurveys carried out in bovine population over 10 years ago revealed the presence of specific antibodies in roughly one-fourth of cows [47]. This seroprevalence did not, however, correspond to the apparent status of examined cows which were mostly clinically healthy. IHC confirmed the presence of SRLV p28 capsid antigen in macrophages and bronchial epithelial cells of a representative sample of goats examined in our study. This allows us to assume that interstitial lesions are likely a manifestation of CAE. A negative result and variable intensity of IHC staining may result from the limited expression of viral antigen within the lesion, assay validity [48], fixation technique, and genetic differences of SRLV isolates [49]. Nevertheless, whether the high prevalence of CIP in goats with symptomatic CAE can be attributed solely to SRLV infection or is, at least to some extent, associated with concurrent chronic infections with other pathogens, including *Mycoplasma* spp., needs to be clarified. It must also be stressed that environmental conditions in the herd from which the goats came were poor, including low-quality ventilation. Insufficient ventilation is known to play a principal role in the development of respiratory tract infections [8]. Therefore, the role of environmental infections should also be taken into account in the evaluation of exposure of the study population to respiratory pathogens and the development of pathological lesions in the lungs.

The interstitial lesions observed in our study were mostly mild, and thus unlikely to have a direct impact on the goats’ clinical condition. More important is the fact that we revealed a statistically significant positive association between CIP and the two other types of pneumonia—PBP and FPP, which are usually of bacterial etiology [8]. Furthermore, we observed a significantly higher prevalence of the infection with potential etiological agents of bacterial pneumonia in goats with CIP. These observations may imply that CIP creates favorable conditions for the concurrent development of other pulmonary pathologies and infections. This observation appears to correspond to the phenomena observed in the course of maedi-visna: the mortality rate is usually low in uncomplicated cases; however, maedi-visna disease places sheep at an increased risk of secondary pulmonary infections which directly contribute to their death [29,50,51]. Nevertheless, pathophysiological studies investigating the ability of CIP to facilitate the development of other pneumonias and the potential molecular mechanism of such a relationship are lacking. Although SRLV is closely related to immunodeficiency lentiviruses (human, feline, bovine), it has never been shown to have any immunosuppressive potential [52,53]. In fact, long-lasting infection overstimulates the immune system, leading to the development of local inflammatory processes and tissue destruction [54,55]. In the organs such as lungs or mammary gland which have direct contact with the external environment, this could create a favorable background for the multiplication of pathogens inhabiting the respiratory tract, teat skin, or canal. The potential existence of this relationship has recently been suggested in terms of the mammary gland of goats with asymptomatic SRLV infection [56]. Obviously, such conclusions need to be drawn very cautiously from cross-sectional studies. Their design, in which both the disease and factors potentially contributing to its occurrence are being detected in animals at the same moment, precludes identifying which of them has emerged first. The knowledge of a right time sequence between the two phenomena (i.e., that exposure to the supposed cause precedes the disease) is essential to consider the relationship between them as causal [57]. In our situation, we may only expect that the goats enrolled in the study had been infected with SRLV since the first months of their life because SRLV spreads efficiently via both lactogenic and horizontal routes [58,59] and the infection had been widespread in the herd long before the goats were born. Moreover, goats in this herd were kept in a relatively small building which ensures their close and intensive mutual contact. We do not know, however, whether it was CIP or any of the two other pneumonia types that developed first. Therefore, our causal inferences should be considered as preliminary results which warrant further confirmation in prospective longitudinal studies.

## 4. Materials and Methods

### 4.1. Goats and Serological Testing

The cross-sectional study was carried out in the years 2016–2018 on female dairy goats of the Polish White Improved and Polish Fawn Improved breeds. The goats came from a large herd (roughly 500 dairy goats) located in western Poland. The herd practiced machine milking twice a day and were used to sell milk to a large dairy. Goats in this herd were housed in a concrete, former piggery building, with a low ceiling and poor gravity ventilation. Goats were kept on straw bedding and were not grazed on the pasture. Main forages were hay, haylage, or corn silage, and main concentrates were wheat and oat.

CAE was first diagnosed in the herd serologically in 2002 in a male goat purchased from another herd. It spread gradually, and, at the beginning of the 2010s, the within-herd seroprevalence approached 100% and the symptomatic form of CAE became common. SRLV circulating in the herd belonged to genotype A and its nucleotide sequence was the closest to the subtype A2 or A3 with 91% identity according to the Basic Local Alignment Search Tool (BLAST) of the National Center for Biotechnology Information (NCBI) [60]. Since the emergence of the first clinical cases of CAE, no regular control program had been in practice in this herd except for regular culling of goats severely affected by clinical signs indicative of CAE. Goats selected by the farmer for culling due to severe arthritis or emaciation were transported to the Warsaw Institute of Veterinary Medicine and humanely euthanized with pentobarbital sodium at a dose of 100 mg/kg i.v. (Morbital, Biowet Puławy, Poland) preceded by a deep general anesthesia with intramuscular mixture of ketamine (Ketamina, Biowet Puławy, Puławy, Poland) at a dose of 5 mg/kg and xylazine (Xylapan, Vetoquinol Biowet Sp. z o.o., Gorzów Wielkopolski, Poland) at a dose of 1 mg/kg. During euthanasia, blood was collected from the jugular vein into 10 mL dry tubes and left at +4 °C overnight for clotting. Then, the blood samples were centrifuged and tested serologically for SRLV using two indirect ELISAs—ELISA based of the whole virus antigen (ID Screen MVV/CAEV Indirect; ID.vet, Grabels, France) and ELISA based on recombinant transmembrane (TM) and capsid (CA) antigens (IDEXX MVV/CAEV p28 Ab Screening, IDEXX Laboratories, Westbrook, ME, USA). According to the manufacturers’ manuals, the following cut-off values were applied in testing: a sample-to-positive control ratio (S/P%) > 50% and S/P% > 110%, respectively. At these cut-off values, the assays’ diagnostic sensitivities (Se) were 92% and 99%, respectively, whereas their diagnostic specificities (Sp) were 92% and 97%, respectively [61,62]. The goats were included in further analyses only if they tested positive in both ELISAs (serial testing) to maximize the probability that they were truly infected with SRLV. The serum samples from these goats were also tested serologically for the presence of antibodies against phospholipase D produced by *C. pseudotuberculosis* in the course of CLA using ELITEST CLA ELISA (Hyphen Biomed, Neuville-sur-Oise, France) and for PIV-3 infection using a quantitative ELISA (Goat PIV-IgG ELISA Kit, ABclonal Technology, Woburn, USA). Both tests were performed according to the manufacturers’ manuals. The PIV-3 ELISA measured the concentration of IgG class antibodies to PIV-3 (ng/mL) and the cut-off value to classify a goat as seropositive was set at >7.5 ng/mL based on a previous study [63]. Blood collection was approved by the 3rd Local Ethics Committee in Warsaw (approval No. 31/2013). According to Polish legal regulations (the Act on the Protection of Animals Used for Scientific or Educational Purposes of 15 January 2015), no ethics committee permission was necessary for post-mortem examination.

### 4.2. Gross Examination

The necropsies of 122 goats were performed within one hour of death. Lungs were examined visually and by palpation in search for gross features suggestive of SRLV-associated pneumonia such as failure to collapse, enlargement, presence of rib impressions on the costal surface of the lungs, diffuse firmness, as well as dryness of cross-sections, and the presence of 1–2 mm white-grey granular patches in the parenchyma [50,64]. From each goat, a fragment of lung tissue 3 cm × 3 cm × 2 cm was collected from the ventral part of the cranial right pulmonary lobe, the one considered as most often affected by pneumonia [8], and fixed in the 10% buffered formalin for histopathological examination. Additionally, a bronchial swab from the tertiary bronchi from the same region was collected for bacteriological examination.

### 4.3. Histopathological Examination

After formalin fixation of the lung samples, they were embedded in paraffin (Paraplast, WITKO Group, Łódź, Poland) and cut at 3.0 µm of thickness using a rotational microtome. Then, specimens were routinely processed and stained with hematoxylin and eosin (H-E; Varistain Gemini, Thermo Fisher Scientific, Waltham, MA, USA), using Masson’s method to demonstrate collagen fibers of the connective tissue and Weigert’s method to demonstrate the fibrin.

The following lesions observed in the histopathological examination were subjectively assessed by a board-certified veterinary pathologist in terms of their severity as mild (1 point), moderate (2 points), and severe (3 points), and assigned to three types of pneumonia:

- Chronic interstitial pneumonia (CIP) composed of six lesions [29,50,64,65,66]: (1) lymphatic nodules hyperplasia (peribronchial and perivascular); (2) interstitial (i.e., peribronchiolar and perialveolar) inflammatory infiltrates composed of lymphocytes, plasma cells, and macrophages; (3) interstitial connective tissue hyperplasia; (4) interalveolar septum thickening; (5) smooth muscle hyperplasia in the walls of the bronchioles; and (6) pneumocyte hyperplasia.
- Purulent bronchopneumonia (PBP) composed of six lesions: (1) peribronchial inflammatory infiltrates mostly composed of neutrophils and some lymphocytes and plasma cells; (2) perivascular inflammatory infiltrates mainly composed of neutrophils, some lymphocytes, and plasma cells; (3) exudate rich in the neutrophils in the lumen of alveoli; (4) focal emphysema; (5) focal atelectasis; and (6) focal necrosis of lung parenchyma.
- Fibrinous pleuropneumonia (FPP) composed of three lesions: (1) interstitial accumulation of fibrin, (2) subpleural accumulation of fibrin, and (3) subpleural connective tissue hyperplasia.

Congestion and lung edema were not included in the classification as all goats were pharmacologically sedated and euthanized, which is likely to be associated with congestion of alveolar capillaries and alteration of vascular permeability in the lungs [67].

For each type of pneumonia, a numerical score was calculated as the sum of points divided by the number of lesions making up the entire type (i.e., the average number of points). The score took values from 0 to 3 and was interpreted as follows based on former classifications in sheep [30]: 0–0.4, no HP lesions; >0.4–1.4, mild; >1.4–2.4, moderate; and >2.4, severe.

Lung specimens from 30 randomly selected goats were further evaluated by an immunohistochemistry (IHC) assay to detect SRLV p28 capsid antigen [48]. After dewaxing in xylene and rehydration in alcohols, the slides were microwaved in 0.02 M citrate buffer, pH 6.0, at 600 Watt for 12 min and then treated with 3% hydrogen dioxide solution for 10 min. The sections were incubated with the 1:20-diluted anti-CAEV (primary) antibody (MAb CAEV-63, CAEV-Co IgG1, CAEP10A1, VMRD, Pullman, WA, USA) overnight at 4 °C in a humid chamber [31]. After washing in TRIS-buffered saline, the sections were incubated with the 1:150-diluted polyclonal goat anti-mouse immunoglobulins conjugated with horseradish peroxidase (secondary antibody) (Gt a Mo immunoglobulins/HRP, P044701-2, Dako, Agilent Tech, Santa Clara, CA, USA), and then visualized with 3,3′-diaminobenzidine (DAB) (Liquid DAB+, K346811-2, Dako, Agilent Tech., Santa Clara, CA, USA). The sections were counterstained with Mayer’s hematoxylin (Htx, Mayers, S330930-2, Dako, Agilent Tech., Santa Clara, CA, USA) and mounted using the DPX medium (Sigma-Aldrich Co., St. Louis, MO, USA). Each IHC stain was performed in parallel with known SRLV-negative caprine control lung tissue (two goats seronegative to SRLV, from a herd free from SRLV infection) and an isotype-control antibody as the negative control (Mo IgG1, Negative Control, X093101-2, Dako, Agilent Tech, Santa Clara, CA, USA).

### 4.4. Bacteriological Examination

Bronchial swabs were delivered to the microbiology laboratory within 2 h of collection and inoculated on two Columbia agar plates supplemented with 5% sheep blood and one MacConkey agar plate (Graso Biotech, Starogard Gdański, Poland). One Columbia agar and the MacConkey agar plate were incubated for 48 h at 37 °C in aerobic conditions. The other Columbia agar plate was cultured for 72 h at 37 °C in microaerophilic conditions. The obtained isolates were initially characterized based on their colony and cell morphology. The identification of isolates to the species level was performed using an adequate API system (bioMérieux, Craponne, France) according to the manufacturer’s instructions. The following bacterial species were considered as potential etiological agents of bacterial pneumonia [8,68,69]: *M. hemolytica*, *P. multocida*, *T. pyogenes*, *S. aureus*, α-hemolytic streptococci, and *C. pseudotuberculosis* as an etiological agent of CLA. Other bacteria were considered as accidental/commensal microorganisms.

### 4.5. Statistical Analysis

Categorical variables were expressed as counts and percentages of animals in a group, and the 95% confidence intervals (CI 95%) for proportions were calculated using the Wilson score method [70]. The relationships between factors were analyzed using a maximum likelihood G test and reported as a prevalence ratio (PR) with CI 95% [57]. Numerical variables were presented as the median, interquartile range (IQR), and range, and compared between groups using a Mann–Whitney U test. All tests were two-sided. The significance level (α) was set at 0.05. Statistical analysis was performed in TIBCO Statistica 13.3 (TIBCO Software Inc., Palo Alto, CA, USA).

## 5. Conclusions

Mild to moderate microscopic features of CIP are present in roughly 50% to 60% of goats with advanced arthritis and emaciation caused by CAE. Histopathological features of other types of pneumonia occur significantly more often in goats with CIP. The causal character of this association should be considered and verified in prospective longitudinal studies.

## Figures and Tables

**Table 1:** The occurrence of histopathological lesions in the lungs of goats with symptomatic caprine arthritis-encephalitis (CAE).

Histopathological Lesion		*N* (%) of Goats with a Lesion	*n* (% of *N*) of Goats with a Lesion of Particular Severity		
			Mild	Moderate	Severe
**Chronic Interstitial Pneumonia (CIP)**					
1	Interstitial inflammatory infiltrate composed of macrophages and lymphocytes	101 (87.1)	72 (71.3)	17 (16.8)	12 (11.9)
2	Interstitial connective tissue hyperplasia	72 (62.1)	48 (66.7)	14 (19.4)	10 (13.9)
3	Lymphoid follicles formation	69 (59.5)	51 (73.9)	14 (20.3)	4 (5.8)
4	Smooth muscle hyperplasia	34 (29.3)	32 (94.2)	1 (2.9)	1 (2.9)
5	Interstitial septum thickening	16 (13.8)	14 (87.5)	0 (0)	2 (12.5)
6	Pneumocyte hyperplasia	6 (5.2)	3 (50.0)	2 (33.3)	1 (16.7)
**Purulent Bronchopneumonia (PBP)**					
1	Peribronchial inflammatory infiltrate composed of neutrophils and lymphocytes	114 (98.3)	85 (74.6)	16 (14.0)	13 (11.4)
2	Perivascular inflammatory infiltrate composed of neutrophils and lymphocytes	95 (81.9)	77 (81.1)	6 (6.3)	12 (12.6)
3	Emphysema	26 (22.4)	26 (100)	0 (0)	0 (0)
4	Bronchial exudate	17 (14.7)	9 (52.9)	1 (5.9)	7 (41.2)
5	Atelectasis	13 (11.2)	12 (92.3)	1 (7.7)	0 (0)
6	Necrosis of lung parenchyma	10 (8.6)	9 (90.0)	1 (10.0)	0 (0)
**Fibrinous Pleuropneumonia (FPP)**					
1	Subpleural connective tissue hyperplasia	68 (58.6)	53 (77.9)	8 (11.8)	7 (10.3)
2	Subpleural fibrin accumulation	27 (23.3)	27 (100)	0 (0)	0 (0)
3	Interstitial fibrin accumulation	15 (12.9)	14 (93.3)	0 (0)	1 (6.7)

**Table 2:** The occurrence of bacteria in the lungs of symptomatic small ruminant lentivirus (SRLV)-seropositive goats.

Bacterial Species	No. of Goats Infected	Prevalence (CI 95%)
**Gram-Positive**		
*Trueperella pyogenes*	16	13.8 (8.7–21.2)
*Staphylococcus* spp.	9	7.8 (4.1–14.1)
*Staphylococcus aureus*	8	6.9 (3.5–13.0)
Coagulase-negative staphylococci	1	0.9 (0.2–4.7)
Others		
*Enterococcus* sp.	9	7.8 (4.1–14.1)
α-hemolytic *Streptococcus* sp.	6	5.2 (2.4–10.8)
*Corynebacterium pseudotuberculosis*	6	5.2 (2.4–10.8)
**Gram-Negative**		
Enterobacterales	24	20.7 (14.3–28.9)
*Escherichia coli*	20	16.8 (11.2–24.5)
*Proteus* sp.	3	2.6 (0.9–7.3)
*Enterobacter* sp.	1	0.9 (0.2–4.7)
Pasteurellaceae	14	11.8 (7.1–18.8)
*Mannheimia hemolytica*	8	6.7 (3.4–12.7)
*Pasteurella multocida*	6	5.2 (2.4–10.8)
Others		
*Pseudomonas aeruginosa*	3	2.6 (0.9–7.3)
*Alcaligenes* sp.	1	0.9 (0.2–4.7)
*Acinetobacter* sp.	1	0.9 (0.2–4.7)

**Table 3:** Chronic interstitial pneumonia (CIP) as a risk factor for other respiratory system-associated conditions.

Respiratory System-Associated Conditions	CIP (*n* (% of *N*))		Prevalence Ratio (CI 95%)	*p*-Value
	Present (*N* = 67)	Absent (*N* = 49)		
Purulent bronchopneumonia (PBP)	39 (58.2)	9 (18.4)	3.17 (1.70–5.92)	<0.001
Fibrinous pleuropneumonia (FPP)	30 (44.8)	8 (16.3)	4.16 (1.69–10.2)	0.001
Infection with potential etiological agents of bacterial pneumonia	28 (41.8)	12 (24.5)	1.71 (0.97–3.01)	0.050
*Corynebacterium pseudotuberculosis* infection	3 (4.5)	3 (6.1)	0.73 (0.15–3.47)	0.696
Presence of accidental/commensal microorganisms	18 (26.9)	15 (30.6)	0.88 (0.49–1.56)	0.659
Antibodies to *Corynebacterium pseudotuberculosis* phospholipase D antigen	38 (56.7)	32 (65.3)	0.87 (0.65–1.16)	0.349
Concentration of antibodies to parainfluenza virus type 3 (PIV-3) (ng/mL)	257, 173–540 (75–1494)	296, 146–53 (39–1454)	–	0.898

## Data Availability

Data are available upon request from the corresponding authors.
